# Fuzzy cognitive mapping in participatory research and decision making: a practice review

**DOI:** 10.1186/s13690-024-01303-7

**Published:** 2024-05-20

**Authors:** Iván Sarmiento, Anne Cockcroft, Anna Dion, Loubna Belaid, Hilah Silver, Katherine Pizarro, Juan Pimentel, Elyse Tratt, Lashanda Skerritt, Mona Z. Ghadirian, Marie-Catherine Gagnon-Dufresne, Neil Andersson

**Affiliations:** 1 Department of Family Medicine, McGill University, 5858 Ch. de la Côte-des-Neiges, Montreal, QC H3S 1Z1 Canada; 2 Universidad del Rosario, Grupo de Estudios en Sistemas Tradicionales de Salud, Bogota, Colombia; 3 Facultad de Medicina, Universidad de La Sabana, Chía, Colombia; 4 Institut Lady Davis pour la Recherche Médicale, Montreal, Canada; 5 École de santé publique, Département de médecine sociale et préventive, Université de Montréal, Montreal, Canada; 6 Centro de Investigación de Enfermedades Tropicales, Universidad Autónoma de Guerrero, Acapulco, Mexico

**Keywords:** Fuzzy cognitive mapping, Participatory modelling, Weight of evidence, Stakeholder engagement, Fuzzy logic, Public health, Global health

## Abstract

**Background:**

Fuzzy cognitive mapping (FCM) is a graphic technique to describe causal understanding in a wide range of applications. This practice review summarises the experience of a group of participatory research specialists and trainees who used FCM to include stakeholder views in addressing health challenges. From a meeting of the research group, this practice review reports 25 experiences with FCM in nine countries between 2016 and 2023.

**Results:**

The methods, challenges and adjustments focus on participatory research practice. FCM portrayed multiple sources of knowledge: stakeholder knowledge, systematic reviews of literature, and survey data. Methodological advances included techniques to contrast and combine maps from different sources using Bayesian procedures, protocols to enhance the quality of data collection, and tools to facilitate analysis. Summary graphs communicating FCM findings sacrificed detail but facilitated stakeholder discussion of the most important relationships. We used maps not as predictive models but to surface and share perspectives of how change could happen and to inform dialogue. Analysis included simple manual techniques and sophisticated computer-based solutions. A wide range of experience in initiating, drawing, analysing, and communicating the maps illustrates FCM flexibility for different contexts and skill bases.

**Conclusions:**

A strong core procedure can contribute to more robust applications of the technique while adapting FCM for different research settings. Decision-making often involves choices between plausible interventions in a context of uncertainty and multiple possible answers to the same question. FCM offers systematic and traceable ways to document, contrast and sometimes to combine perspectives, incorporating stakeholder experience and causal models to inform decision-making. Different depths of FCM analysis open opportunities for applying the technique in skill-limited settings.

**Supplementary Information:**

The online version contains supplementary material available at 10.1186/s13690-024-01303-7.

## Background

Collaborative generation of knowledge recognises people’s right to be involved in decisions that shape their lives [1]. Their participation makes research and interventions more relevant to local context and priorities and, thus, more likely to be effective [2]. A commitment to the co-creation of knowledge proposes that people make better decisions when they have the benefit of both scientific and other forms of knowledge. These include context-specific understanding, knowledge claims based on local settings, experience and practice, and organisational know-how [3]. Participatory research expands the idea of what counts as evidence, opening space for the experience and knowledge of stakeholders [4, 5]. The challenge is how to create a level playing field where diverse knowledges can contribute equally. We present fuzzy cognitive mapping (FCM) as a rigorous and transparent tool to combine different perspectives into composite theories to guide shared decision-making [6–8].

In the early 1980s, the combination of fuzzy logic [9] to concept mapping of decision making [10, 11] led to FCM [12]. Fuzzy cognitive maps are directed graphs [13] where nodes correspond to factors or concepts, and arrows describe directed influences. Using this basic structure for causal relationships, users can represent their knowledge of complex systems, including many interacting concepts. Many variables are not easily measured or estimated with precision or are hard to circumscribe within a formal definition, for example, wellbeing, cultural safety, or racism [14, 15]. Nevertheless, their causes and effects are important to capture for decision-making. Fuzzy cognitive maps offer a formal structure to include these kinds of variables in the analysis of complex health issues.

The flexibility of the technique allows for systematic mapping of knowledge from multiple sources to identify influences on a particular outcome while supporting collective learning and decision making [16]. FCM has been used across multiple fields with applications that include modelling, prediction, monitoring, decision-making, and management [17–20]. FCM has been applied in medicine to aid diagnosis and treatment decision-making [21, 22]. FCM has also supported community and stakeholder engagement in environmental sciences [23, 24] and health by examining conventional and Indigenous understanding of causes of diabetes [25].

Many implementation details contribute to interpretability of FCM, a common concern for researchers new to the technique. This review addresses these practical details when we used FCM to include local stakeholder understanding of causes of health issues in co-design of actions to tackle those issues. The focus is on transparent mapping of stakeholder experience and how it meets requirements for trustworthy data collection and initial analysis. The methods section describes what fuzzy cognitive maps are and how we documented our experience using them. We describe tools and procedures for researchers using FCM to incorporate different knowledges in health research. The results summarize experience in four stages of mapping: framing the outcome of concern, drawing the maps, performing basic analyses, and using the resulting maps. The discussion contrasts our practices with those described in the literature, identifying potential limitations and suggesting future directions.

## Methods of the practice review

**Fig. 1:**
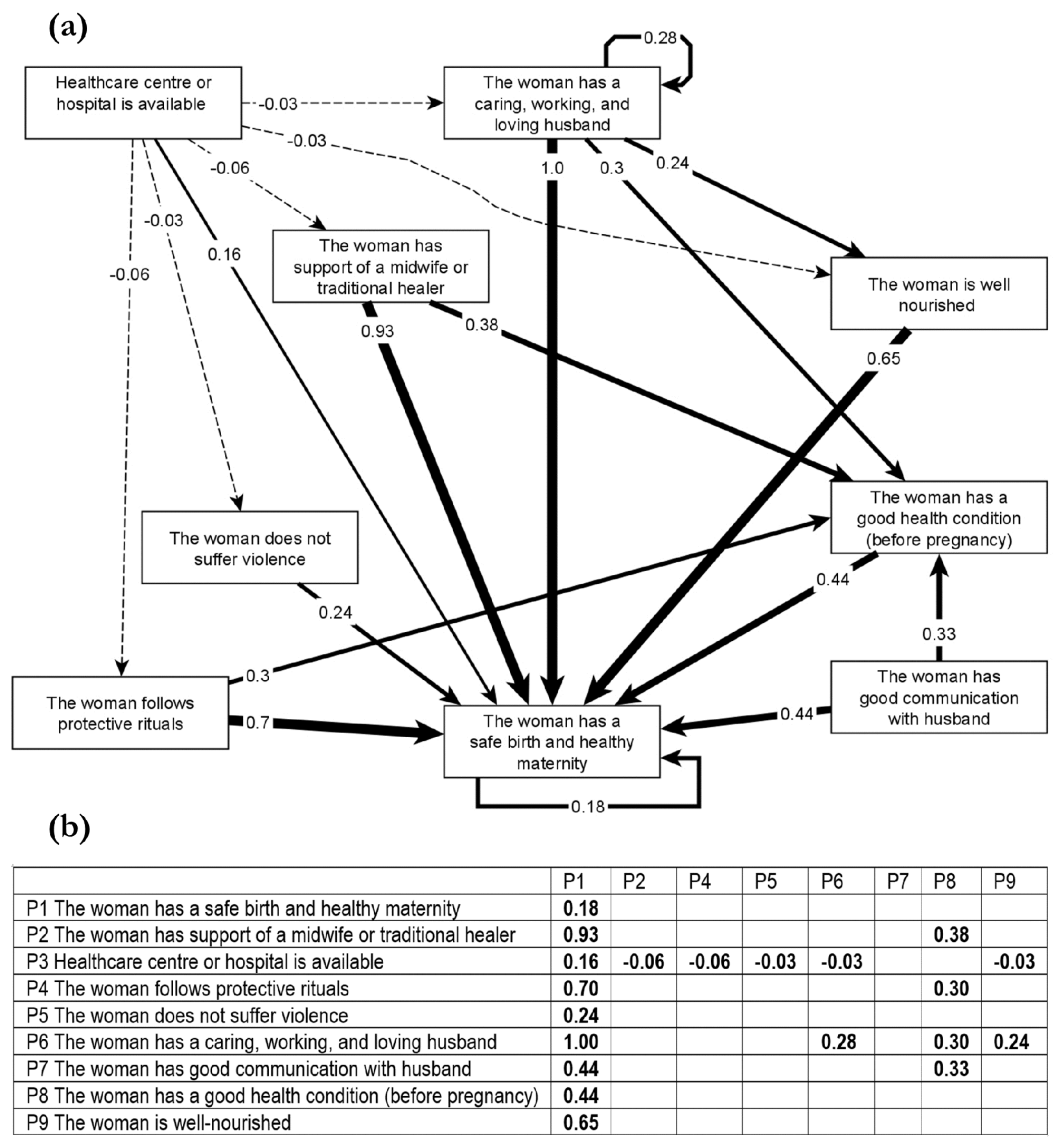
Fuzzy cognitive map of causes of a healthy maternity according to indigenous traditional midwives in Guerrero, Mexico. (**a**) Graphical display of a fuzzy cognitive map. The boxes are nodes, and the arrows are directed edges. Strong lines indicate positive influences, and dashed lines indicate negative influences. Thicker lines correspond to stronger effects. (**b**) Adjacency matrix with the same content as the map. Rows and columns correspond to the nodes. The value in each cell indicates the strength of the influence of one node (row) on another (column). Reproduced without changes with permission from the authors of [26]

Fuzzy cognitive maps are graphs of causal understanding [6]. The unit of meaning in fuzzy cognitive mapping is a relationship, which corresponds to two nodes (concepts) linked by an arrow. Arrows originate in the causes and point to their outcomes. A cause can lead to an outcome directly or through multiple pathways (succession of arrows). Figure 1 shows a fuzzy cognitive map of causes of healthy maternity according to indigenous traditional midwives in the South of Mexico [26].

The “fuzzy” appellation refers to weights that indicate the strength of relationships between concepts. For example, a numeric scale with values between one and five might correspond to *very low*, *low*, *medium*, *high* or *very high* influence. If the value is 0, there is no causal relationship, and the concepts are independent. Negative weights indicate a causal decrease in the outcome, and positive weights indicate a causal increase in the outcome. A tabular display of the map, an adjacency matrix, has the concepts in columns and rows. The value in a cell indicates the weight of the influence of the row concept on the column concept (Fig. 1). A map can also be represented as an edge list. This shows relationships across three columns: causes (originating node), outcomes (landing node) and weights. Some maps use ranges of variability for the weights (grey fuzzy cognitive maps) [27] or fuzzy scales to indicate changing states of factors [21].

Following rules of logical inference, the relationships between concepts can suggest potential explanations for how they work together to influence a specific outcome [28, 29]. One might interpret a cognitive map as a series of *if-then* rules [9] describing causal relationships between concepts [12]. For example, *if* the quality of health care increases, *then* the population’s health should also improve. Maps can incorporate feedback loops [30], such as: *if* violence increases, *then* more violence happens.

An international participatory research group met in Montreal, Canada, to share FCM experience and discussed its application. FCM implementation in all cases shared a common ten-step protocol [6], with results of almost all exercises published in peer-reviewed journals. The lead author of each publication presented their work and corroborated the synthesis reflected the most important aspects of their experiences. A webpage details the methods, materials, and tools members of the group have used in practice (https://ciet.org/fcm).

As a multilevel training exercise, the meeting included graduate students, emerging researchers with their first research projects and experienced FCM researchers. Nine researchers presented their experience, challenges and lessons learned. The senior co-author (NA) led a four-round nominal group discussion covering consecutive mapping stages: (1) who defined the research issue and how, (2) procedures for building maps and the role of participants at each point, (3) analysis tools and methods and (4) use of the maps. Before the session, participants received the published papers concerning the FCM projects under discussion and the guiding questions about the four themes. After the meeting, the first author (IS) transcribed and drew on the session recording to draft the manuscript. All authors subsequently contributed to the manuscript, which follows the approach used to describe our work with narrative evaluations [31]. The summary of FCM methods used, the results of the practice review, follows the categories used in the nominal group to inquire about FCM implementation.

## Results

Researchers reported their practice in three different FCM applications. Most cases mapped stakeholder knowledge in the context of participatory research [26, 32–38,39]. They also described using FCM to contextualise mixed-methods literature reviews in stakeholder perspectives [5, 40, 41, 42] and to conduct secondary quantitative analysis of surveys [43–45]. A fourth FCM application, not discussed in detail in this paper, is in graduate teaching. A master’s program in Colombia and a PhD course in Canada incorporated the creation of cognitive maps as a learning tool, with each student building a map to describe how their research project could contribute to promoting change.

Table 1 summarises the characteristics of the 25 FCM practices reviewed. The number of maps varied from a handful to dozens. Table 2 summarises the processes of defining the issue, drawing, analysing, and using the three different kinds of maps: stakeholder knowledge, mixed-methods literature reviews, and questionnaire data. Table 3 summarises the FCM processes in each of the four mapping stages. Of 23 FCM publications from the group since 2017 (see Additional File 1), four describe methodological contributions [4–6, 35], and the rest describe the use of FCM in specific contexts.

**Table 1:** Characteristics of the discussed experiences in the application of FCM

Case	Summary	Country and year	Number of maps and participants
1. Behavioural change in a dengue prevention trial in Mexico [43]	A secondary analysis of a cluster randomised controlled trial modelled intermediate results between knowing about dengue prevention and taking actions for dengue prevention	Mexico, 2016	Four maps stratified by gender and intervention status, 6185 participants
2. Culturally safe attitudes in a medical education trial in Colombia [44, 45]	A secondary analysis of a randomised controlled trial with medical students modelled intermediate results between knowing about cultural safety and behavioural change	Colombia, 2019	Two maps stratified by intervention status, 347 participants
3. Synthesis of a scoping review on maternal health in Indigenous communities with traditional midwives [39, 40]	Fuzzy cognitive maps summarised the results of a scoping review and allowed a comparison between the literature and stakeholder maps	The Americas, 2020	One literature map summarised 87 studies
4. Evacuation of Inuit mothers from Nunavik	Literature maps showing factors contributing to and resulting from medical evacuation for maternal health among Inuit women in Nunavik	Canada, 2019	One literature map summarised 61 studies
5. Pilot study to develop techniques and approaches around the *Weight of Evidence* [4, 5]	The maps represented unmet postpartum care needs of immigrant women in Canada from both the literature and stakeholders to develop the Weight of Evidence procedure	Canada, 2019	One literature map and three stakeholder maps, 5 participants
6. Experience of judgement of pregnant and parenting adolescents in Ottawa [34]	Maps helped to summarise and discuss with stakeholders the results of a mixed-methods literature review on perinatal outcomes and experiences of adolescents during pregnancy to identify priority areas of concern	Canada, 2020	A literature map and 10 stakeholder maps, 10 participants
7. Adolescent parents contextualised evidence about factors contributing to child protection involvement [78]	Maps represented summarised evidence about factors contributing to child protection involvement among adolescent parents, which were then adjusted and prioritised by adolescent parents and shared with child protection workers	Canada, 2020	A literature map and 10 stakeholder maps, 10 participants
8. Indigenous knowledge of risk and protective factors for maternal health [26]	The experience is part of an overarching project to promote safe birth in cultural safety among indigenous communities. The maps summarised the knowledge of senior traditional midwives	Mexico, 2017	Four stakeholder maps, 29 participants
9. Views of intercultural researchers on risk and protective factors for maternal health [35]	The maps contrasted perspectives of stakeholders and developed methods for the application of Harris’s discourse analysis	Mexico, 2018	Eight stakeholder maps, eight participants
10. Causes and protectors for short childbirth interval (*kunika*) in Northern Nigeria [33]	The maps summarised community knowledge of actionable factors to address *kunika* and informed co-design of communication materials around *kunika* for use in home visits to pregnant women and their spouses	Nigeria, 2018	52 stakeholder maps, over 400 participants
11. Causes and protectors for short childbirth interval in Northern Uganda [79]	The maps were part of a mixed-methods study to inform culturally safe strategies to promote child spacing in Northern Uganda	Uganda, 2019	21 stakeholder maps, 168 participants
12. Barriers and facilitators of access to perinatal health care [80]	Part of a mixed-methods design, the maps aimed to identify local stakeholder views of facilitators and barriers to accessing perinatal health care	Uganda, 2019	21 stakeholder maps, 168 participants
13. Explore the possible implementation of HPV self-sampling in Nunavik [32]	The maps facilitated identifying Inuit women’s views of what needs to change to improve HPV screening in their communities	Canada, 2018	Ten stakeholder maps, 27 participants
14. Access of young women to Government services and support programs	Maps of young women and service providers allowed to have two sides of the story to explain why women do not use official services and programs	Botswana, 2016	11 stakeholder maps, 66 participants
15. Violence against young women [36, 38]	The maps portrayed community stakeholder views of causes of violence; they supported updating of a docudrama in an intervention to reduce violence	Botswana, 2020	12 stakeholder maps, 68 participants
16. Suicide among young men [37, 38]	The maps portrayed community stakeholder views of factors contributing to suicide among young men	Botswana, 2021	Nine stakeholder maps,46 participants
17. Men’s and women’s wellbeing in Indigenous communities	FCM involved communities in understanding contributors to men’s and women’s wellbeing to identify possible interventions with an emphasis on strength-based actions	Guatemala, 2018	40 stakeholder maps, 265 participants
18. Birthing in a good way for Inuit women in Nunavik	Stakeholder maps will contribute to building a theory of what would contribute to good childbirth among women in Nunavik	Canada, 2021 to 2023	86 stakeholder maps, 117 participants
19. Balanced diet and iron-rich for adolescent girls in rural Ghana [42]	In the context of a randomised trial, mapping sessions helped adolescent girls to identify and reflect on pressing issues that they would address in a co-designed video intervention	Ghana, 2020	20 stakeholder maps, 181 participants
20. Satisfaction with HIV care [81]	Women living with HIV shared their experiential expertise to adjust literature maps on factors contributing to satisfaction with HIV care	Canada, 2021	24 stakeholder maps, 23 participants and one literature map
21. Sexual & reproductive health of adolescents [82]	Communities in Northern Nigeria share their views on factors influencing adolescent sexual & reproductive health	Nigeria, 2022	77 stakeholder maps, 312 participants
22. Participation of urban communities from low- and middle-income countries in public and global health research [41]	Fuzzy cognitive mapping summarised the results of a scoping review, which will be contextualised in the views of local stakeholders in Dhaka, Bangladesh, to develop recommendations for the implementation of a cluster randomised controlled trial on dengue	Bangladesh, 2023	1 literature map summarises 85 studies. Stakeholder will create additional maps
23. Moose habitat	FCM of factors contributing to the quality of moose habitat in Eeyou Istchee, Northern Quebec, according to indigenous perspectives	Canada, 2022	35 maps, 56 participants
24. Causes of academic fraud among medical students	Students and faculty create maps on their views of the causes of academic fraud to inform deliberative dialogue on potential actions	Colombia, 2022	5 maps, 25 participants
25. Intercultural healthcare in Colombia	Senior Indigenous leaders share their perspectives on what could contribute to increasing intercultural healthcare in Colombia	Colombia, 2023	25 maps, 25 participants

**Table 2:** Summary of the FCM processes and use of the maps for stakeholder maps, literature maps and maps from questionnaire data

Knowledge source	How the issue was defined and who participated	Drawing the maps	Analysis methods	How the maps were used
Stakeholders (Cases 5 to 25)	– Multiple mechanisms to define the focus: researcher or stakeholder lead based on literature reviews; a direct request from communities [83]; discussions with communities, religious leaders [84] or advisory boards [32] based on local evidence; and funding agency [79, 80] or public authority lead – Diverse participants in terms of age, gender, cultural backgrounds and urban or rural contexts	– Individual or group sessions facilitated by trained community members or junior researchers – Mapping sessions followed predefined protocols – In-person or online sessions – Some communities experienced challenges weighting relationships	– Pattern correspondence table – Transitive closure and cumulative net influence – Harris’ discourse analysis – Realist analysis – Social network analysis – Thematic analysis and reduction	– Grounding and contextualising evidence in stakeholder views – Methods development – Co-design of interventions – Discussion with stakeholders using simplified maps – Scientific papers
Mixed-methods literature reviews (Cases 3 to 5 and 22)	– Part of overarching participatory research with previously defined objectives – Literature maps helped to identify relevant issues that community members prioritised – Included quantitative, qualitative and mixed-methods studies – Scoping and systematic reviews of published and grey literature	– The maps represent relationships reported in empirical studies – For systematic reviews, map weights derived from the mathematical transformation of odds ratios and other effect measures into the range (-1 to + 1) – For scoping reviews, weights arose from the reported relationship in the literature and Harris’s discourse analysis	– Pattern correspondence table – Transitive closure and cumulative net influence – Harris’s discourse analysis – Realist analysis – Social network analysis – Thematic analysis and reduction	– Grounding and contextualising evidence in stakeholder views – Methods development – Scientific papers
Questionnaire data (Cases 1 and 2)	– Part of overarching participatory research with previously defined objectives – Secondary analysis of questionnaire data – Aggregated individual questionnaires of urban and rural populations with diverse cultural backgrounds – FCM was used as an analysis method and did not require ethical approval	– Maps with seven nodes corresponding to seven intermediate outcomes of planned behaviour change (CASCADA) – Weights arose from the mathematical transformation of odds ratios into the range (-1 to + 1)	– Transitive closure and cumulative net influence – Comparison of subgroups	– Impact assessment of an intervention – Scientific papers

**Table 3:** Summary of processes used in the four stages of FCM in the reported cases

Objective	FCM steps	Tools www.ciet.org/fcm
1. **Initiating the mapping process**		
Develop the focus and obtain ethical review	– It requires other participatory research techniques before mapping – Includes ethical assessments by institutional review boards or stakeholder representatives	– Guiding questions to initiate FCM – Script to obtain oral informed consent – Formats and videos to describe a mapping session with stakeholders
Engage and contrast relevant sources of knowledge to extend the understanding of the issue	– Identify participants and data sources across three possible applications of FCM: questionnaire data, literature reviews and stakeholder maps	
2. **Drawing the maps**		
***Stakeholder maps***		
Describe stakeholder knowledge of the causes of health issues	– Groups or individuals share their knowledge about causes, identify duplicates, draw arrows to indicate causal influences and weight the strength of each link	– FCM video – Training materials for facilitators – List of dos and do nots – Spreadsheet for thematic analysis – Free software for drawing the maps – Video on how to digitise maps
***Literature reviews***		
Summarise available literature on the causes of a health outcome. It is part of a broader objective to ground evidence in stakeholder experience	– Described in detail as part of the Weight of evidence procedure – Uses standard methods to retrieve the evidence – Quantitative relationships and qualitative themes reported in the literature are summarised in maps – Measures the strength of the relationships using ORs or other scaled effect measures	– Guide to the WoE – Formula to scale ORs in the range − 1 to 1 – Tools to scale effect measures into ORs
Summarise heterogeneous literature about the causes of a health outcome	– The maps summarise the relationships identified in the scoping reviews – Harris’ discourse analysis returns estimated weights	– Formats for data collection in scoping reviews – Spreadsheet for thematic analysis – Harris’ discourse analysis
***Questionnaire data***		
Generate models from questionnaire data to identify the influences across a chain of intermediate results toward behavioural change	– Creates a list of relationships between **C**onscious knowledge, **A**ttitude, **S**ubjective norm, intention to **C**hange, **A**gency, **D**iscussion and **A**ction – Measures the strength of the relationships using ORs	– CASCADA model – CIETmap to calculate ORs – Formula to scale ORs in the range − 1 to 1
Summarise semi-structured interviews about causal relationships of health issues	– Qualitative semi-structured interviews ask participants about the causes of health issues – A thematic analysis identifies the influences that constitute the map, and discourse analysis calculates the weights	– Spreadsheet for thematic analysis – Harris’ discourse analysis
**3. Analysing the maps**		
Contrast the perspectives of multiple sources	– Pattern correspondence table to compare direct and indirect influences and their weights. It can happen before or after digitisation	– Pattern correspondence table
Digitise maps to allow the computer-based analyses	– Present maps as adjacency matrices or edge lists to facilitate calculations	– yEd or Mental Modeler (free software)
Identify net influences between factors in the maps through direct or indirect paths	– Two models (fuzzy and probabilistic) can be used to calculate transitive closure – The mathematical models also generate a list of direct and indirect relationships	– Free software (ProbTC and FuzzyTC embedded in CIETmap - FCM module)
Combine maps from multiple sources	– Maps can be combined using a simple or weighted average of the relationship across maps. Bayesian procedures allow updating the weights across multiple maps.	– CIETmap – FCM module – Algorithms: TC of combined maps (by Mateja Šajna)
Identify the importance of concepts in the map	– Measures of social network analysis use incoming and outgoing arrows to identify the nodes that work mostly as causes or as outcomes	– yEd, Mental Modeler, R scripts
Generate category-level maps to summarise stakeholder perspectives	– Creates maps by thematically grouping factors with similar meanings – A mathematical procedure calculates category-level influence weights	– FCM video – Algorithms: aggregation of maps (by Mateja Šajna)
Identify explanatory accounts and framework related to the issue	– Realist analysis of maps uses the *if-then* rules linking nodes across different relationships together with stakeholder narrative accounts and supplementary literature review	– WoE Guide
**4. Using the maps**		
Identify stakeholder priorities and theories of change	– Discussion of local evidence from maps and other sources contributes to identifying priority solutions – Edit and summarise maps to communicate their key contents clearly	– Summary maps for communication

### Stage 1. Who defined the issue and how

Focus group discussions or conversations with partners were the most common methods for defining the issue to be mapped. Cases #6 (pregnant and parenting adolescents) and #20 (women’s satisfaction with HIV care) used literature maps to identify priorities with participants in Canada, while cases #5 (immigrant’s unmet postpartum care needs) and #7 (child protection involvement) contextualised literature-based maps with stakeholder knowledge. In cases #15 and #16 on violence against women and suicide among men in Botswana, community members involved in another project raised these issues as concerns. Two cases used FCM in the secondary analysis of survey data to answer questions defined by the research teams (#1 Mexico dengue) and academic groups (#2 Colombia medical education).

All cases used a participatory research framework [46]. FCM worked both in well-established partnerships (#8 and #9 involved researchers and Indigenous communities in Mexico, and #20 well-established partnerships with women living with HIV) and in the early stages of trust building (#6 adolescent parents in Canada).

#### Ethics

Almost all cases reported two levels of ethical review: institutional boards linked with universities and local entities (health ministries and authorities, advisory boards, community organisations or leaders). Most review boards were unfamiliar with FCM, and some requested additional descriptions and protocols to help them understand the method. In Guatemala (#17) and Nunavik (#18), Indigenous authorities and a steering committee requested a mapping session themselves before approving the project. Most projects used oral consent, mainly due to the involvement of participants with a wide range of literacy levels and in contexts of mistrust about potential misuse of signed documents (Indigenous groups in #8) or during virtual mapping sessions (women living with HIV in #20).

#### Strengths-based or problem-focused

Most cases followed a strengths-based approach, focusing on what influences a positive outcome (for example, what causes good maternal health instead of what causes maternal morbidity or mortality). Some cases created two maps: one about causes of a positive outcome and one about causes of the corresponding negative outcome (#8 causes and risks for safe birth in Indigenous communities, and #10 causes and protectors of short birth interval). Building two maps helped to unearth additional actionable concepts but was time-consuming and tiring for the stakeholders creating the maps.

#### Broad concepts or tight questions

A recurring issue was how broad the question or focus should be. A broad question about ‘what influences wellbeing’ fitted well with the holistic perspectives of Mayan communities but posed challenges for drawing, analysing, and communicating maps with many concepts and interactions (#17, Guatemala). A very narrowly defined outcome, on the other hand, might miss potentially actionable causes.

### Stage 2. Drawing maps

In the group’s experience, most people readily understand how to make maps, given their basic structure (cause, arrow and consequence). Based on their collective experience, the research group developed a protocol to increase replicability and data quality in FCM, particularly for stakeholder maps, which often involve multiple facilitators and different languages. Creating maps from literature reviews and questionnaire data did not have some of the complications of creating maps with stakeholders but also benefitted from detailed protocols.

#### Stakeholder maps

The mapping cases reviewed here included mappers ranging from highly trained university researchers (#9 on safe birth) to people without education and speaking only their local language (#8 in Mexico, #10 and #21 in Nigeria, #11 and #12 in Uganda). Meeting participants discussed the advantages and disadvantages of group and individual maps. Groups stimulate the emergence of ideas but include the challenge of ensuring all participants are heard. Careful training of facilitators and managing the mapping sessions as nominal groups helped to increase the participation of quieter people. Groups of not more than five mappers were much easier to facilitate without losing the creative turbulence of a group. Most cases relied on small homogeneous groups, run separately by age and gender, to avoid power imbalances among the map authors. Individual sessions worked well for sensitive topics. They accommodated schedules of busy participants and worked for mappers not linked to a specific community.

**Fig. 2:**
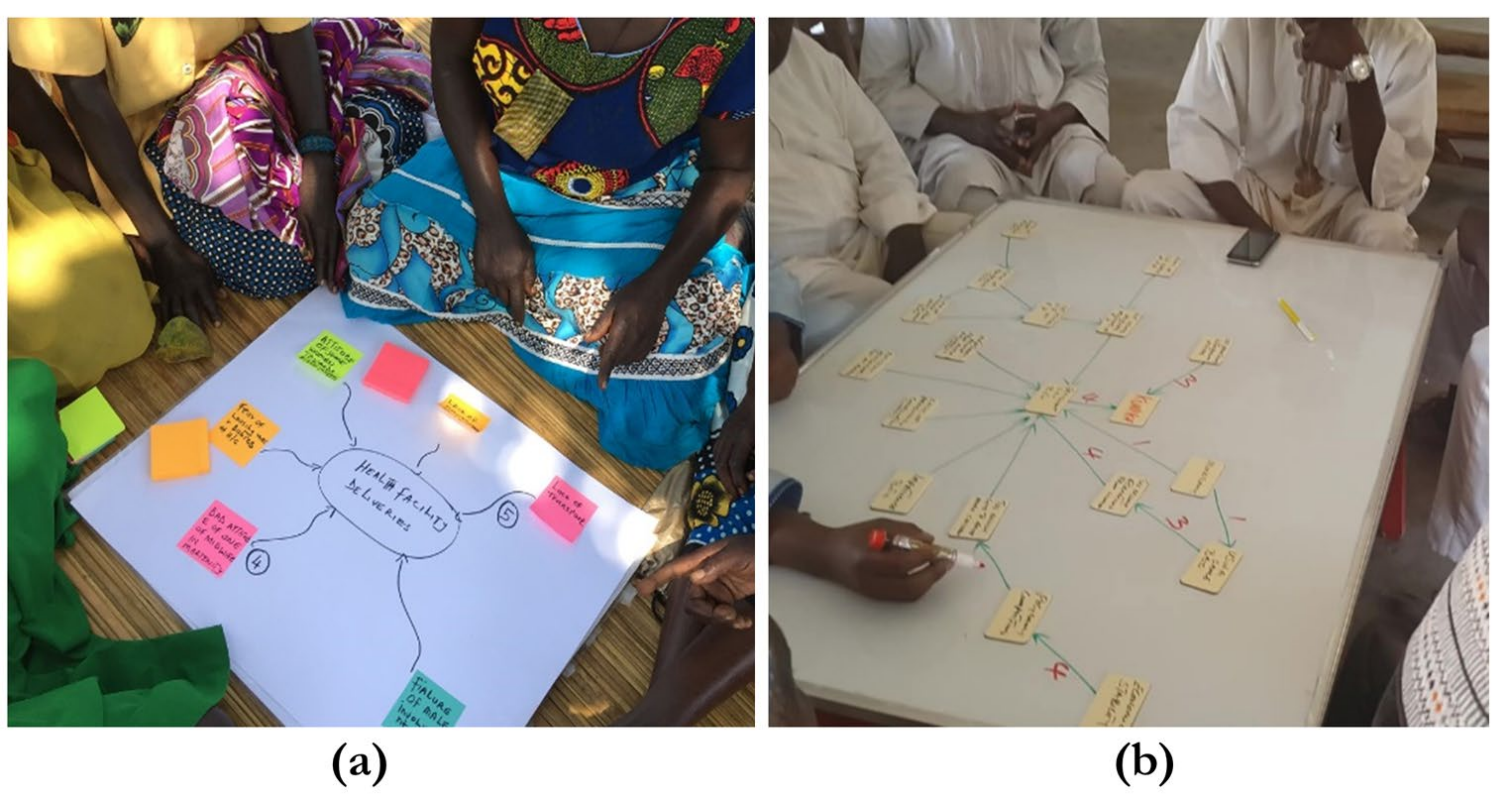
Fuzzy cognitive maps from group sessions in Uganda and Nigeria. (**a**) A group of women in Uganda discusses what contributes to increasing institutional childbirths in rural communities. They used sticky notes and markers on white paper to draw the maps. (**b**) A group of men in Northern Nigeria uses a whiteboard and magnetic tiles to draw a map on causes of short birth intervals

Basic equipment for mapping is inexpensive and almost universally available. Most researchers in our group used either sticky notes on a large sheet of paper or magnetic tiles on a metal whiteboard (Fig. 2). Some researchers had worked directly with free software to draw the electronic maps (www.mentalmodeler.com or www.yworks.com/products/yed), while others digitised the physical maps, often from a photograph. Three cases conducted FCM over the internet or telephone, with individual mappers (#9, #20 and #25) constructing their maps online in real-time.

Group mapping sessions typically had a facilitator and a reporter to take notes on the discussions. Reporters are crucial in recording explanations about the meaning of concepts and links. Experienced researchers stressed that careful training of facilitators and reporters, including several rounds of field practice, is essential to ensure quality. We developed materials to support training and quality control of mapping sessions (#21 Nigeria), available at www.ciet.org/fcm. In Nigeria (#21), the research team successfully field-tested the use of Zoom technology via mobile handsets with internet connection by the cellular network to allow virtual participation of international researchers in FCM sessions in the classroom and communities.

Many mappers in community groups had limited or no schooling and only verbal use of their local language. It worked well in these cases for the facilitators to write the concepts on the labels in English or Spanish, while the discussion was in the local language. Facilitators frequently reminded the groups about the labels of the concepts in the local language. In case #16 in Botswana, more literate groups wrote the concepts in Setswana, and the facilitators later translated them into English. Most researchers found that the FCM graphical format helped to overcome language barriers, and it seems to have worked equally well with literate and illiterate groups. Additional file 2 lists common pitfalls and potential solutions during group mapping sessions.

#### Identifying causes of the issue

Some mapping sessions started by asking participants what the central issue of the map meant to them. This was useful for comparing participant views about the main topic (#8 and #9 maternal health in Indigenous communities and #20 satisfaction with HIV care) and in understanding local concepts of broad topics (#17 Indigenous wellbeing). In Nigeria (#21), group discussions defined elements of adolescent sexual and reproductive health before undertaking FCM, and facilitators shared the list of elements with participants in mapping sessions. In Nunavik (#13 Canada, Inuit women on HPV self-sampling), participating women received an initial presentation to create a common understanding to discuss HPV self-sampling, an unfamiliar technique in Inuit communities.

Some cases created stakeholder maps from scratch, asking participants what they thought would cause the main outcome (#8 to 10, 14 to 19, 21, and 23 to 25). Other cases reviewed the literature first and presented the findings to participants (#5, 7 and 20). In these cases, the facilitators reminded participants that literature maps might not represent their experiences. They encouraged them to add, remove and reorganise concepts, relationships, and weights until they felt the map represented their knowledge.

Once participants had identified concepts (nodes), facilitators had to carefully consider the wording of the labels to represent the meaning of each node and identify potential duplicates. They confirmed duplications with participants and removed repeated nodes. In case #19, participating girls first had one-on-one conversations to discuss and prioritise what they thought contributed to a balanced diet. In a second activity, the actual mapping session, participants organised those concepts into categories and voted on their priorities for action. The Nigerian cases, with large numbers of maps, included creation of an iterative list of labels, with new concepts added after each mapping session to ensure the use of standard labels in future sessions when the mappers confirmed that the standard label wording indicated what they wanted to convey. This step is helpful in the combination of maps that we describe in stage 3.

#### Drawing arrows

Some maps showed mainly direct influences on the central issue, while others identified multiple relationships between concepts in the map. When the central issue was too broad, participants found it hard to assign relationships between concepts (#17). Facilitators frequently asked participants to clarify the meaning of proposed causal pathways or how they perceived one factor would lead to another and to the main outcome (see Additional file 2). To ensure arrows were appropriately labelled as positive or negative, some facilitators used standardised *if-then* questions to draw the relationships. For example, if factor A increases, does factor B increase or decrease? (#9).

#### Weighing

All the presented cases used a scale from one to five to indicate the weights of links. Many Indigenous participants insisted that all the concepts were equally important (#8, 13 and 18). Careful training of facilitators encouraged participant weighing (#10, 15 and 16). It was often helpful to identify the two relationships with extreme upper and lower weights and use those as a reference to weight the rest of the relationships.

#### Verifying the maps

Stakeholder sessions ended with a verification of the final map. This initial member checking preceded any additional analysis. Participants readily accepted the technique and reported satisfaction that they could see concrete representations of their knowledge by the end of the FCM sessions (#13). It reaffirmed what they knew and what they could contribute in a meaningful way. In Ghana (#19 adolescent nutrition), young participants described mapping sessions as empowering when interviewed six months later [42].

#### Synthesis of literature reviews

FCM can portray qualitative and quantitative evidence from the literature in the same terms as stakeholder experience and beliefs and is a cornerstone of an innovative and systematic approach called the Weight of Evidence. In this approach, stakeholders interpret, expand on, and prioritise evidence from literature reviews (#5 unmet postpartum care needs [5, 34], #3 maternal health in communities with traditional midwives [40], #4 medical evacuation of Indigenous pregnant women [47], #7 child protection investigations among adolescent parents, and #22 community participation in health research) [41].

Case #5 (Weight of Evidence) demonstrated how to convert quantitative effect estimates (e.g., odds ratio, relative risk) into a shared format to facilitate comparison between findings [5]. When multiple effect estimates described the same relationship, appropriate techniques [7, 48, 49] allowed for calculating pooled estimates. In #5, qualitative concepts represented ‘unattached’ nodes when the studies suggested they contributed to the outcome of interest. The researchers updated the literature maps with stakeholder views using a Bayesian hierarchical random-effects model with non-informative priors [50].

In scoping reviews with a broader topic and more heterogeneity of sources (#3, #22) [40], the map reported the relationships and their supporting data, such as quotes for qualitative studies and odds ratios for quantitative ones, instead of unifying the results in a single scale. Each relationship was counted as 1 (present) with positive or negative signs. Data extraction used a predefined format in which at least two independent researchers registered the relationships after reading the full texts. Each included study contributed to the model in the same way it would contribute to an overall discourse about the topic.

#### Maps from questionnaire data

**Fig. 3:**
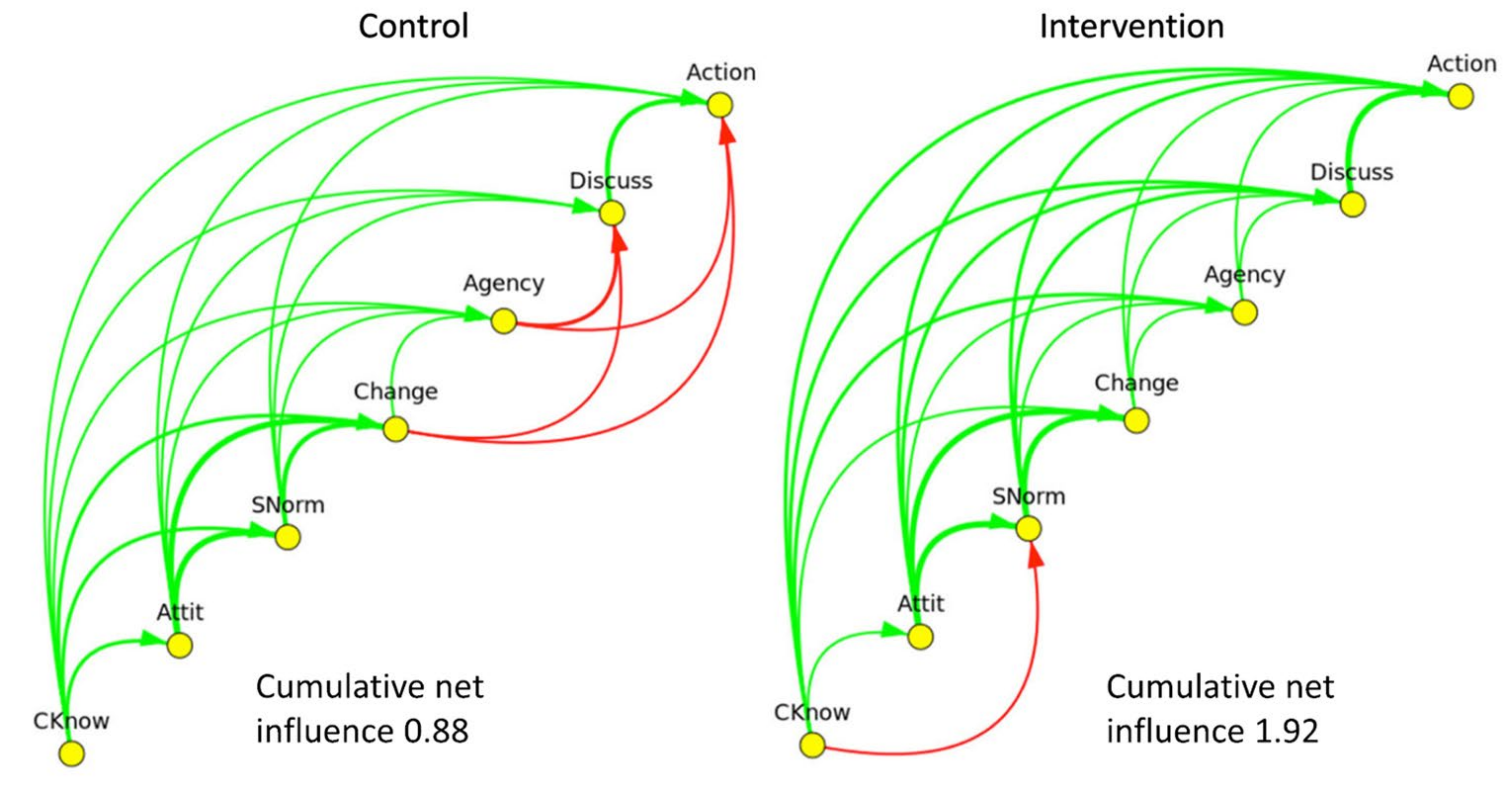
Maps from questionnaire data from the study on dengue control in Guerrero, Mexico. Green arrows are positive influences, and red arrows correspond to negative influences. The control group showed a negative influence in the results chain with a cumulative net influence of 0.88; the intervention group showed no such block and a cumulative net influence of 1.92. Reproduced without changes with permission from the authors of [43]

Researchers used questionnaire data to generate maps of a behavioural change model in dengue prevention in Mexico [43] and cultural safety among medical trainees in Colombia [44, 45]. The dengue project produced separate maps for men and women, while the Colombian map included all participants. Each map had seven nodes, one for each domain of change in the CASCADA model of behavioural change (Fig. 3): Conscious knowledge, Attitudes, positive deviation from Subjective norms, intentions to Change behaviour, Agency, Discussion of possible action and Action or change of practice [51]The surveys included questions for each intermediate result, and the repeat survey during the impact assessment provided a counterfactual comparison. For example, in Mexico (#1), Conscious knowledge (first C) was the ability to identify a physical sample of a mosquito larva during the interview, and Action (last A) focused on participation in collective activities in the neighbourhood to control mosquito breeding sites. The maps in Colombia (#2) explored the CASCADA network of partial results towards the students’ self-reported intention to change their patient-related behaviour.

The arrows linking the nodes received a weight (*w*) equivalent to the odds ratio (OR) between the outcomes, transformed to a symmetrical range (-1 to 1) using the formula proposed by Šajna:

$$w =1-\left(\frac{2}{OR+1}\right)$$

### Stage 3. Tools and methods to analyse the maps

#### Comparing levels of influence

Initial analysis of maps includes a pattern correspondence table that lists and contrasts direct and indirect influences reported from different sources. Free software allows for digitising maps and converting them into lists of relationships or matrices for more complex analyses. In our analysis approach, we first calculate the transitive closure (TC) of each map. This mathematical model provides the total influence of one concept on all others after considering all the possible paths linking them [7]. Two models are available [52]: fuzzy TC, recommended for maps with ad hoc concepts, and probabilistic TC, often used for maps with predetermined concepts. With the transitive closure of a map, it is possible to build a pattern correspondence table comparing influences according to different knowledge sources. Table 4 shows an example.

**Table 4:** Example pattern correspondence table contrasting the three strongest influences on adequate childbirth spacing by stakeholder groups in Uganda

	Service providers	Men	Traditional midwives	Community health workers	Women	Male youths	Female youths
Sufficient material resources	1.00	1.00	0.56	1.00	1.00	1.00	0.39
Mothers and children healthy	0.33	0.40	1.00	0.28	0.06	0.43	0.17
Desire to have a better life	0.42	0.30	0.24	0.04	0.13	0.23	0.39

The values in the table correspond to category-level weights according to each stakeholder group. The scale is 0.00 to 1.00, with 1.00 as the strongest influence. Reproduced without modifications with authorisation of the authors of [73,79]

Additional tools for analysing the maps include centrality scores from social network analysis. These measures compare the sum of the absolute values of the weights of incoming or outgoing edges to identify the total importance of a node [53]. Higher levels of out-degree centrality indicate more influence on other concepts, and higher values of in-degree centrality suggest that the concepts are important outcomes in the map [16].

#### Operator-independent weighting

In response to the challenges of participant weighting in some contexts, we applied Harris’ discourse analysis to calculate overall weights across multiple maps based on the frequency of each relationship across the whole discourse (e.g., multiple maps from stakeholders or studies in literature reviews). Harris intended to have an operator-independent alternative to identify the role of morphemes (part of a word, a word or several words with an irreducible meaning) in a discourse, exclusively from their occurrence in the text [54]. Because it used frequency, among other criteria (partial order, redundancies and dependencies), it did not depend on the researcher’s assumptions of meaning. Similarly, we intended to understand the causal meaning of relationships identified through FCM with an operator-independent procedure. A concept that caused an outcome across multiple maps would have a stronger causal role than a concept that caused the same outcome only in one or two maps. We found that analysis of maps using discourse analysis and participant weighting produced similar results [35].

#### Combining maps

In many cases, the analysis included bringing the transitive closure maps together as an average representation of stakeholder groups. Combining maps often required reconciling differences in labels across maps. This was also an opportunity to generate categories to describe groups of related factors. Some cases involved stakeholders in this process, while others applied systematic researcher-led procedures followed by member checking exercises to confirm categories. Combining maps used weighted or unweighted averages of each relationship’s weight across maps. It also used stakeholder-assigned Bayesian priors to update corresponding relationships identified in the literature [5].

#### Reduction of maps

Stakeholder and literature maps usually have many factors and relationships, making their analysis complex and hindering communication of results. We created reduced maps following a qualitative synthesis of nodes and a mathematical procedure to calculate category level weights [35]. Some maps in Canada have engaged participants in defining the categories as they progress with the mapping session (#7). However, creating categories within individual mapping sessions can lead to difficulties with comparability between groups when the categorisation varies between them.

#### Sensemaking of relationships

Weighting by stakeholders helps prioritise direct and indirect influences that contribute to an outcome. Stakeholder narratives and weights helped to develop explanations of how different factors contribute to the outcomes. In cases #5 and #7, an additional literature search based on factors identified by stakeholders contributed to creating explanatory accounts. The reporting of women’s satisfaction with HIV care (#20) used quotes recorded in the mapping sessions to explain the narratives of the most meaningful relationships. The analysis of maps on violence against women in Botswana (#15) identified important intermediate factors commonly depicted along the pathways from other factors to the main outcome.

### Stage 4. How maps were used

Researchers described how they edited and simplified complex maps to make them more accessible, including to people with limited literacy, in Mexico, Nigeria, and Uganda (#8 and 10 to 12). In addition to creating category maps, they used colour coding, labels in the local language for the most influential factors, arrows of different thicknesses according to their weight, and different sizes of boxes for concepts according to their importance based on centrality scores. When sharing results, they often contrasted maps from different stakeholders. In Canada (#5 and #7), researchers developed explanatory frameworks from the mapping exercises, and stakeholders refined this framework and identified priority areas for action. In Canada (#5 and #7), Botswana (#14) and Uganda (#11 and #12), stakeholders viewed and discussed the summary maps from other groups. The maps, further discussed by stakeholders, helped inform the design of media-based communication interventions in Ghana (#19) and Nigeria (#10).

## Discussion

Our experiences with FCM resonate with and adds considerable detail to earlier FCM authors [18, 19], including those offering protocols for meaningful participation in environmental sciences [16, 49]. The most recent literature reviews on the use of FCM have not discussed the contributions we described here [20, 22, 55, 56] and do not provide details on practical decisions across the mapping process or on the implications of stakeholder authorship. This review provides practical insights for FCM researchers before they generate maps, during data collection and in analysis. The use of FCM to increase data sources in the coproduction of knowledge brings numerous challenges and multiple potential decisions. This paper summarises how we approached these challenges across 25 real-world projects and responds to the questions we often receive from researchers new to the method. These methodological considerations are essential to increase trustworthiness of FCM applications and for an adequate interpretation of its results.

Variability in facilitation of mapping sessions with stakeholders is well recognised as a source of potential differences between groups [19]. In our experience, the behaviour and attitudes of researchers and facilitators can influence the content of the maps. Careful quality control and member checking can help minimise this influence [57]. To achieve high-quality, informative maps, our experience highlights the need for clear protocols for data collection, including careful training of facilitators and ongoing supervision and monitoring. This has been essential in some of our projects, which have involved hundreds of participants in creating hundreds of maps.

Our group also used FCM in contextualising mixed-methods literature reviews. Knowledge synthesis is seldom free of reviewer interpretations [58], and formal protocols for data collection, analysis, synthesis, and presentation could increase the reliability and validity of findings [59]. Singer et al. also used FCM to summarise qualitative data [60], a promising application that benefits from FCM’s *if-then* configurations and linguistic descriptions of concepts and relationships. In our practice, FCM was a practical support to develop formal protocols, to generate pooled effect estimates across studies [58] and to summarising heterogeneous sources. Weight of Evidence is an innovation to incorporate stakeholder perspectives with scientific evidence, thus addressing the common challenge of contextualising literature findings with local realities. The application of FCM in modelling questionnaire data helps to evaluate result chains as knowledge networks.

Despite its name (fuzzy) and tolerance for uncertainty, FCM is not fuzzy or vague [61]. It incorporates multiple dimensions of decision-making, including impressions, feelings, and inclinations, in addition to careful reasoning of events and possibilities [9, 62]. FCM is a participatory modelling approach [63] that improves conventional modelling with real-world experience. FCM can help formalise stakeholder knowledge and support learning about an issue to promote action [64]. An important part of the literature focuses on applying learning algorithms for scenario planning [55, 65]. Our group reported positive changes and increased agency among mappers. Future research might explore the impact of FCM as an intervention, both on those sharing their knowledge and on those using the models. The commitment to operator-independent procedures has led us to adapt Harris’s discourse analysis to complement the sometimes-problematic weighing step [35]. Notwithstanding our ability to generate operator-independent weights, the question of whose views the models represent and who is empowered remains valid and should be discussed in every case [66].

There is very little literature on FCM in education. FCM could help students clarify the knowledge they share in class [67]. FCM can also formalise steps to connect and evaluate students’ progression towards concrete learning objectives, a helpful feature in game-based learning [68]. In our experience, mapping sessions had a transformative effect as participants reflected on what they knew about the main issue and appreciated their knowledge being presented in a tangible product. Further studies could investigate how group and individual characteristics evolve throughout the mapping process.

Decision-making involves choosing alternatives based on their expected impacts. Many people think of FCM in the context of predictive models using learning algorithms [55, 56, 69–72]. There is also potential for informing other AI-driven methods by incorporating expert knowledge in the form of fuzzy cognitive maps into complex graph-based models [73, 74]. The concern in participatory research and, therefore, use of FCM in participatory research, is equitable engagement in informed decision making. We used FCM not as a predictive tool but for making sense of scenarios and theories to inform choices, recognising multiple possible ways of seeing any issue. Map interpretation hinges on who the authors are and the type of data depicted (opinions, observations, or components of a theory. These soft models characterise direct and indirect dependencies that are difficult to incorporate in formal approaches like differential equations [28]. Current work of the research group explores participant-led FCM weighting to inform Bayesian analysis of quantitative data and ethnographic approaches to understand deeper meanings of factors depicted in FCM.

A potential concern about FCM is whether the sample size and selection are adequate, yet FCM reports rarely discuss this. There are no formal procedures to estimate the required sample size for mapping exercises (total number of participants, maps, or people in a group session). Singh and Chudasama, for example, continued mapping sessions until the list of causal factors identified reached saturation [75]. A participatory research approach, however, would conduct as many mapping sessions as much as necessary to allow all voices, especially those of the most marginalised, to be heard. Our application of Harris’ discourse analysis allows quicker mapping sessions, avoiding the often lengthy weighting process; this can increase the number of maps that can be created with finite resources. The combination of maps results in more robust models because more knowledge informs the final output [76]. Multiple alternatives exist for combining maps [5, 8, 21, 77]. Our work has explored Bayesian updating using stakeholder weights as priors [5].

## Strengths and limitations

Almost all the experiences described in this review are published and provide further details on specific topics. This practice review reflects the experience in participatory research and thus mainly focused on stakeholder maps. Our group pioneered the use of FCM for contextualising systematic reviews in stakeholder experience. We also used FCM to analyse and to portray progress in changing a results chain in a modified theory of planned behaviour. Operator bias is a constant concern in our FCM practice, reflected in the review of efforts to avoid operator influence in generating the maps, in the coding of map concepts into categories, and especially in weighting of maps, where our innovation relies on Harris’ discourse analysis.

The general use of FCM has well-recognised challenges and limitations. It is easy to forget that cognitive maps reflect opinions and personal experience, which can differ between map authors and from biological causality. This is seldom a major problem in our participatory research practice, where we frame FCM as different perspectives to engage stakeholders or as an entry point to dialogue. As with most visual techniques, the maps are static and do not model the longitudinal evolution of the depicted knowledge network. Viewers might assume relationships in the maps are linear, which is not always the case [76]. For example, the effect of higher age on maternal health outcomes would be very different for teenagers and older mothers.

Most map readers make inferences from the causes to the outcome, the direction of the arrow not inviting a reversed cause *from* the outcome. Different approaches to causal reasoning could affect map construction, weighting and interpretation; although relatively robust to cultural and educational differences, our experience includes cultural groups that have more complex views of causal relationships than can be reflected in FCM.

Several questions about conducting FCM remain unanswered, such as how to standardise (and limit) the influence of facilitators, how to use FCM with people living with visual or hearing loss, or how to create meaningful maps using distance communication, such as social media, or when participants have limited time for the exercise.

## Conclusions

FCM is a flexible and robust way to share multiple stakeholder perspectives. Although mostly applied to beliefs and experiences, it can also portray published evidence and questionnaire data in formats comparable with subjective experience. FCM requires multiple practical decisions that have implications for interpreting and sharing results. We review these methodological decisions in 25 research projects in different contexts since 2016. Insights might be relevant to researchers interested in using FCM and can contribute to applying it in a more systematic way. Clear protocols and quality control improve the reliability of fuzzy cognitive maps. FCM helps build a shared understanding of an issue across diverse knowledge sources and can provide a systematic and transparent basis for shared decision-making.

### Electronic supplementary material

Below is the link to the electronic supplementary material.

Supplementary Material 1

Supplementary Material 2

## Data Availability

The dataset supporting the conclusions of this article is included within the article and its additional files.
