# Supplementary material for: Fuzzy cognitive mapping in participatory research and decision making: a practice review
Source: Arch Public Health. 2024 May 20;82:76. doi: 10.1186/s13690-024-01303-7 (PMC11103993; doi:10.1186/s13690-024-01303-7)
Supplement: Supplementary file 1 — Supplementary Material 1 [file 13690_2024_1303_MOESM1_ESM.docx]

# Additional file 2. Common challenges when creating maps with stakeholder groups and solutions applied

| **Challenge** | **Description** | **Solution** |
| --- | --- | --- |
| Selection of facilitators | Facilitators are importantly different from the stakeholders (e.g., different ages or languages) | Recruit and train facilitators who can interact easily with the group (e.g., young facilitators for groups of adolescents) |
| The main outcome has different meanings | Participants have different understandings of the meaning of the main outcome | At the start of the session, explain to participants the meaning of the outcome to have a common understanding or ask what it means to them to capture the diversity of definitions |
| Facilitators reproduce their ideas in the maps | Over the course of facilitating maps of several groups, facilitators produce maps which depict their view of the issue as much as the views of the participants | Recognition of the potential problem  Careful training and field practice for facilitators  Supervised field work and debriefing after mapping sessions |
| Poor quality notes | The notes from the mapping sessions do not explain the meaning of concepts, the reasons for linkages, or the reasons for weighing of links | Recognition of the importance of field notes. Protocol for preparing joint fair reports after sessions  Careful training and field practice for reporters  Supervised fieldwork, including review of reports in debriefing sessions |
| Orphan concepts | Some concepts in the map have no direct or indirect paths connecting them to the main outcome | Training facilitators to frequently ask how each factor affects the main issue directly or indirectly  Reporters and supervisors to help identify any orphan concepts during mapping sessions |
| Duplicated concepts | The same concept but with different wording features more than once on the map | Facilitators confirm duplication with participants and eliminate duplicates during mapping sessions  Debriefing sessions identify duplicates and help to avoid future duplicates  Create and use a list of standard labels as the mapping sessions progress |
| Maps have positive and negative expressions of a concept | The positive and negative expression of the same concept features in the map (this is a form of duplication) | Facilitators identify the issue during the mapping and eliminate one expression  Debriefing sessions identify this occurrence and help to avoid it in the future  Create and use a list of standard labels as the mapping sessions progress |
| Arrows in the wrong direction | Instead of describing the causal direction, the arrows point toward the cause | Training includes exercises to identify if-then relationships  Supervised field work and debriefing after mapping sessions |
| Arrows indicate conjunction between factors but not causal relationships | For example, non-use of contraception is linked with an arrow pointing towards frequent sex to indicate that both will contribute to *kunika*. However, non-use of contraception is not the cause of frequent sex | Training includes exercises to identify if-then relationships  Supervised field work and debriefing after mapping sessions |
| All relationships are equally important | Participants say every relationship is equally relevant, and therefore weighting is not possible, or they weigh all links as strength 5 | Encourage participants to identify the weakest and strongest relations and assign weights of 1 and 5, respectively  Fill in other weights or use Harris’ discourse analysis approach as an alternative to participant weighing |
| Confusion of signs and effects | Participants confuse positive and negative weights with good and bad effects | Training includes exercises to identify if-then relationships |
