# Supplementary material for: Fuzzy cognitive mapping in participatory research and decision making: a practice review
Source: Arch Public Health. 2024 May 20;82:76. doi: 10.1186/s13690-024-01303-7 (PMC11103993; doi:10.1186/s13690-024-01303-7)
Supplement: Supplementary file 2 — Supplementary Material 2 [file 13690_2024_1303_MOESM2_ESM.docx]

# Additional file 1. List of publications describing different aspects of our use of FCM

| Year | Reference | Journal |
| --- | --- | --- |
| 2017 | Andersson, N., Beauchamp, M., Nava-Aguilera, E., Paredes-Solís, S., & Šajna, M. (2017). The women made it work: Fuzzy transitive closure of the results chain in a dengue prevention trial in Mexico. BMC Public Health, 17(Suppl 1), 133–173. https://doi.org/10.1186/s12889-017-4301-0 | BMC Public Health |
| 2018 | Dion, A., Joseph, L., Jimenez, V., Gutierrez, A. C., Ben Ameur, A., Robert, E., & Andersson, N. (2019). Grounding evidence in experience to support people-centered health services. International Journal of Public Health, 64(5), 797–802. https://doi.org/10.1007/s00038-018-1180-9 | International J Public Health |
| 2019 | Andersson, N., & Silver, H. (2019). Fuzzy cognitive mapping: An old tool with new uses in nursing research. Journal of Advanced Nursing, 75(12), 3823–3830. https://doi.org/10.1111/jan.14192 | J Advanced Nursing |
| 2020 | Sarmiento, I., Paredes-Solís, S., Loutfi, D., Dion, A., Cockcroft, A., & Andersson, N. (2020). Fuzzy cognitive mapping and soft models of indigenous knowledge on maternal health in Guerrero, Mexico. BMC Medical Research Methodology, 20(1), 125. https://doi.org/10.1186/s12874-020-00998-w | BMC Medical Research Methodology |
| 2020 | Sarmiento, I., Paredes-Solís, S., Morris, M., Pimentel, J., Cockcroft, A., & Andersson, N. (2020). Factors influencing maternal health in indigenous communities with presence of traditional midwifery in the Americas: protocol for a scoping review. *BMJ Open*, *10*(10), e037922. https://doi.org/10.1136/bmjopen-2020-037922 | BMJ Open |
| 2020 | Tratt, E., Sarmiento, I., Gamelin, R., Nayoumealuk, J., Andersson, N., & Brassard, P. (2020). Fuzzy cognitive mapping with Inuit women: what needs to change to improve cervical cancer screening in Nunavik, northern Quebec? BMC Health Services Research, 20(1), 529. https://doi.org/10.1186/s12913-020-05399-9 | BMC Health Services Research |
| 2020 | Belaid, L., Atim, P., Ochola, E., Omara, B., Atim, E., Ogwang, M., Bayo, P., Oola, J., Okello, I. W., Sarmiento, I., Rojas-Rozo, L., Zinszer, K., Zarowsky, C., & Andersson, N. (2021). Community views on short birth interval in Northern Uganda: a participatory grounded theory. Reproductive Health, 18(1), 88. https://doi.org/10.1186/s12978-021-01144-5 | Reproductive Health |
| 2020 | Belaid, L., Atim, P., Atim, E., Ochola, E., Ogwang, M., Bayo, P., Oola, J., Wonyima Okello, I., Sarmiento, I., Rojas-Rozo, L., Zinszer, K., Zarowsky, C., & Andersson, N. (2021). Communities and service providers address access to perinatal care in postconflict Northern Uganda: socialising evidence for participatory action. Family Medicine and Community Health, 9(2), e000610. https://doi.org/10.1136/fmch-2020-000610 | Family Medicine and Community Health |
| 2021 | Sarmiento, I., Ansari, U., Omer, K., Gidado, Y., Baba, M. C., Gamawa, A. I., Andersson, N., & Cockcroft, A. (2021). Causes of short birth interval (kunika) in Bauchi State, Nigeria: systematizing local knowledge with fuzzy cognitive mapping. Reproductive Health, 18(1), 74. https://doi.org/10.1186/s12978-021-01066-2 | Reproductive Health |
| 2021 | Pimentel, J., Cockcroft, A., & Andersson, N. (2021). Game jams for cultural safety training in Colombian medical education: a pilot randomised controlled trial. BMJ Open, 11(5), e042892. https://doi.org/10.1136/bmjopen-2020-042892 | BMJ Open |
| 2021 | Pimentel, J., Cockcroft, A., & Andersson, N. (2021). Impact of game jam learning about cultural safety in Colombian medical education: a randomised controlled trial. BMC Medical Education, 21(1), 132. https://doi.org/10.1186/s12909-021-02545-7 | Medical Education |
| 2021 | Dion A. Chapter 2: The influence of social and economic exclusion on perinatal health outcomes and care experiences in Canada: An integrative review. In: *Broadening what counts as expertise in perinatal health of under-served populations in Canada: participatory methods and Bayesian updating contextualize evidence synthesis in stakeholder knowledge*. Doctoral dissertation. Andersson N, supervisor. McGill University; 2021. https://escholarship.mcgill.ca/concern/theses/02871189q | Reproductive Health |
| 2021 | Dion, A., Klevor, A., Nakajima, A., & Andersson, N. (2021). Evidence‐based priorities of under‐served pregnant and parenting adolescents: addressing inequities through a participatory approach to contextualizing evidence syntheses. International Journal for Equity in Health, 20(1), 118. https://doi.org/10.1186/s12939-021-01458-7 | International J Equity Health |
| 2021 | Ghadirian, M., Marquis, G., Dodoo, N., & Andersson, N. (2022). Ghanaian Female Adolescents Perceived Changes in Nutritional Behaviors and Social Environment After Creating Participatory Videos: A Most Significant Change Evaluation. *Current Developments in Nutrition*, *6*(8), nzac103. https://doi.org/10.1093/cdn/nzac103 | Current Developments in Nutrition |
| 2021 | Sarmiento, I., Paredes-Solís, S., Dion, A., Silver, H., Vargas, E., Cruz, P., Pimentel, J., Zuluaga, G., Cockcroft, A., & Andersson, N. (2021). Maternal health and Indigenous traditional midwives in southern Mexico: contextualisation of a scoping review. BMJ Open, 11(12), e054542. https://doi.org/10.1136/bmjopen-2021-054542 | BMJ Open |
| 2021 | Dion, A., Carini-Gutierrez, A., Jimenez, V., Ben Ameur, A., Robert, E., Joseph, L., & Andersson, N. (2021). *Weight of Evidence*: Participatory Methods and Bayesian Updating to Contextualize Evidence Synthesis in Stakeholders’ Knowledge. Journal of Mixed Methods Research, JMMR-19-03, 155868982110374. https://doi.org/10.1177/15586898211037412 | Journal Mixed Methods Research |
| 2022 | Sarmiento, I., Cockcroft, A., Dion, A., Paredes-Solís, S., De Jesús-García, A., Melendez, D., Marie Chomat, A., Zuluaga, G., Meneses-Rentería, A., & Andersson, N. (2022). Combining Conceptual Frameworks on Maternal Health in Indigenous Communities—Fuzzy Cognitive Mapping Using Participant and Operator-independent Weighting. Field Methods, 34, 1525822X2110704. https://doi.org/10.1177/1525822X211070463 | Field Methods |
| 2022 | Cockcroft, A., Omer, K., Gidado, Y., Mohammed, R., Belaid, L., Ansari, U., Mitchell, C., & Andersson, N. (2022). Impact-Oriented Dialogue for Culturally Safe Adolescent Sexual and Reproductive Health in Bauchi State, Nigeria: Protocol for a Codesigned Pragmatic Cluster Randomized Controlled Trial. JMIR Research Protocols, 11(3), e36060. https://doi.org/10.2196/36060 | JMIR Research Protocols |
| 2022 | Dion, A., Nakajima, A., McGee, A., & Andersson, N. (2022). How Adolescent Mothers Interpret and Prioritize Evidence About Perinatal Child Protection Involvement: Participatory Contextualization of Published Evidence. *Child and Adolescent Social Work Journal*. https://doi.org/10.1007/s10560-022-00865-1 | Adolescent Social Work Journal |
| 2023 | Gagnon-Dufresne, M.-C., Sarmiento, I., Fortin, G., Andersson, N., & Zinszer, K. (2023). Why urban communities from low-income and middle-income countries participate in public and global health research: protocol for a scoping review. BMJ Open, 13(6), e069340. https://doi.org/10.1136/bmjopen-2022-069340 | BMJ Open |
| 2023 | Sarmiento, I., Kgakole, L., Molatlhwa, P., Girish, I., Andersson, N., & Cockcroft, A. (2023). Community perceptions about causes of suicide among young men in Botswana: an analysis based on fuzzy cognitive maps. *Vulnerable Children and Youth Studies*, 1–23. https://doi.org/10.1080/17450128.2023.2262941 | Vulnerable Children and Youth Studies |
| 2023 | Sarmiento, I., Field, M., Kgakole, L., Molatlhwa, P., Girish, I., Andersson, N., & Cockcroft, A. (2023). Community perceptions of causes of violence against young women in Botswana: fuzzy cognitive mapping. *Vulnerable Children and Youth Studies*, 1–57. https://doi.org/10.1080/17450128.2023.2262413 | Vulnerable Children and Youth Studies |
| 2023 | Cockcroft, A., Sarmiento, I., Andersson, N. (2023). Shared perceived causes of suicide among young men and violence against young women offer potential for co-designed solutions: intervention soft-modelling with fuzzy cognitive mapping, *Vulnerable Children and Youth Studies*. https://doi.org/10.1080/17450128.2023.2287623 | Vulnerable Children and Youth Studies |
